# A 42-year-old patient presenting with femoral head migration after hemiarthroplasty performed 22 years earlier: a case report

**DOI:** 10.1186/1752-1947-9-17

**Published:** 2015-01-15

**Authors:** Akio Kanda, Kazuo Kaneko, Osamu Obayashi, Atsuhiko Mogami

**Affiliations:** 16 Department of Orthopedic Surgery, Juntendo Shizuoka Hospital, Izunagaoka 1129, Izunokuni-country 410-2295 Shizuoka, Japan; 17 Department of Orthopedic Surgery, Juntendo University, Hongou 3-1-3, Bunkyou Ward 113-8431 Tokyo, Japan

**Keywords:** Acetabular articular cartilage, Femoral head migration, Femoral neck fracture, Hemiarthroplasty, Infection

## Abstract

**Introduction:**

Treatment of femoral neck fractures in young adults may require total hip arthroplasty or hip hemiarthroplasty using a bipolar cup. The latter can, however, result in migration of the femoral head and poor long-term results.

**Case presentation:**

We report a case of femoral head migration after hemiarthroplasty performed for femoral neck fracture that had occurred 22 years earlier, when the patient (a Japanese man) was 20 years old. He experienced peri-prosthetic fracture of the femur, subsequent migration of the prosthesis, and a massive bone defect of the pelvic side acetabular roof. After bone union of the femoral shaft fracture, the patient was referred to our hospital for reconstruction of the acetabular roof. Intra-operatively, we placed two alloimplants of bone from around the transplanted femoral head into the weight-bearing region of the acetabular roof using an impaction bone graft method. We then implanted an acetabular roof reinforcement plate and a cemented polyethylene cup in the position of the original acetabular cup. Eighteen months post-operatively, X-rays showed union of the transplanted bone.

**Conclusions:**

Treatment of femoral neck fractures in young adults is usually accomplished by osteosynthesis, but it may be complicated by femoral head avascular necrosis or by infection or osteomyelitis. In such cases, once an infection has subsided, either hip hemiarthroplasty using a bipolar cup or total hip arthroplasty may be required. However, if the acetabular side articular cartilage is damaged, a bipolar cup should not be used. Total hip arthroplasty should be performed to prevent migration of the implant.

## Introduction

Treatment of femoral neck fracture in young adults is usually accomplished by osteosynthesis, but treating patients with femoral head avascular necrosis or with infection or osteomyelitis is difficult. After resolution of infection, total hip arthroplasty or hemiarthroplasty of the hip joint using a bipolar cup may be required. However, the latter can result in migration of the femoral head and poor long-term results. We report a case of femoral head migration after hemiarthroplasty performed for femoral neck fracture that had occurred 22 years earlier, when the patient was 20 years old.

## Case presentation

**Figure 1:**
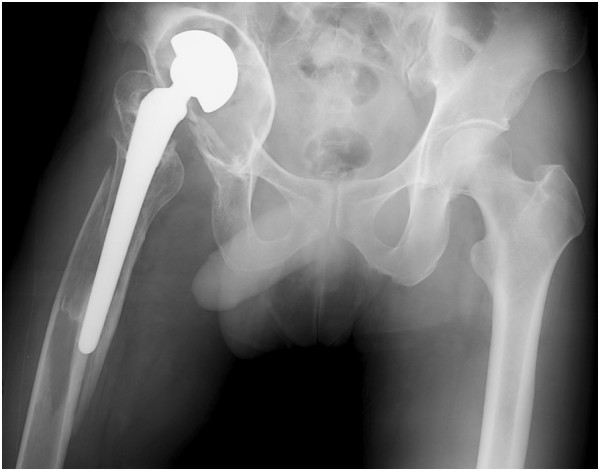
**X-ray showing a peri-prosthetic fracture of the femur.**

**Figure 2:**
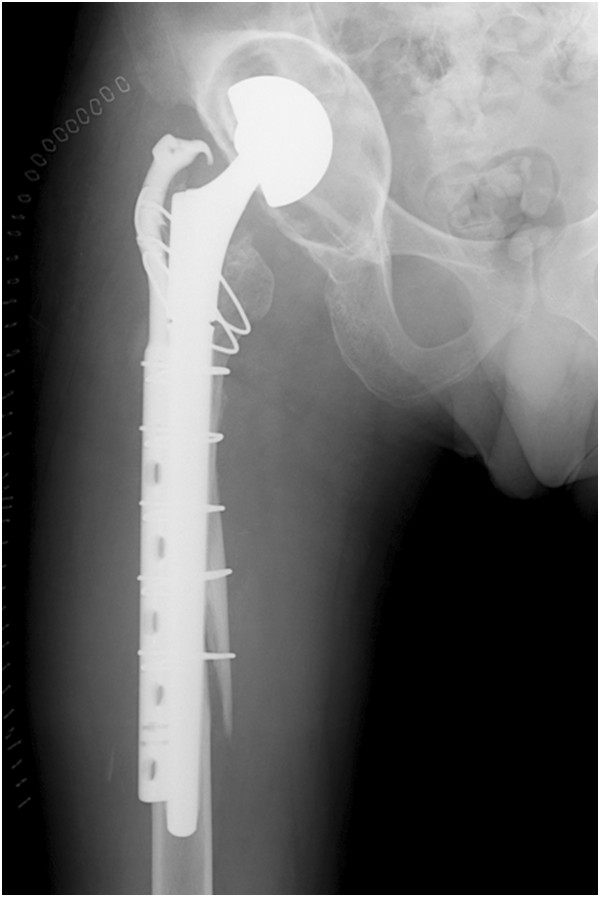
**X-ray showing new hemiarthroplasty of the hip joint with a modular neck and long stem.**

**Figure 3:**
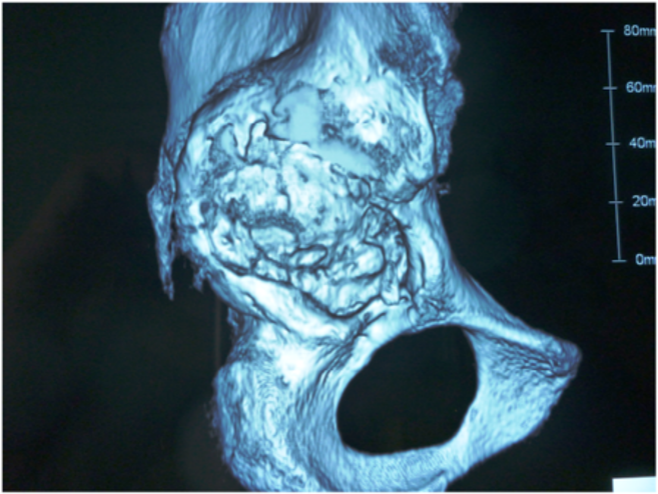
**Three-dimensional computed tomographic scan showing American Academy of Orthopedic Surgeons type II cavitary bone defect of the acetabular roof.**

**Figure 4:**
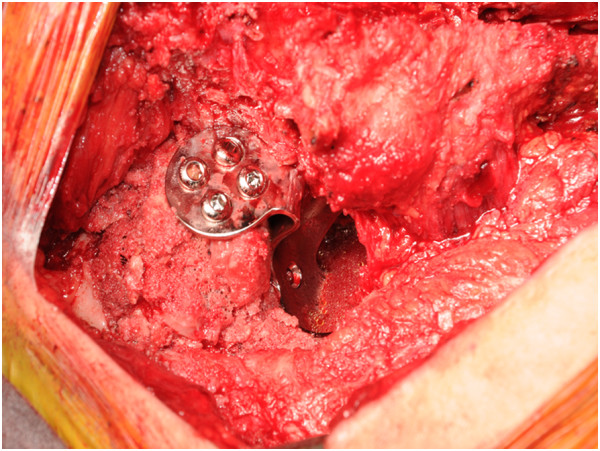
**Intra-operative photograph showing the acetabular roof reinforcement plate.**

**Figure 5:**
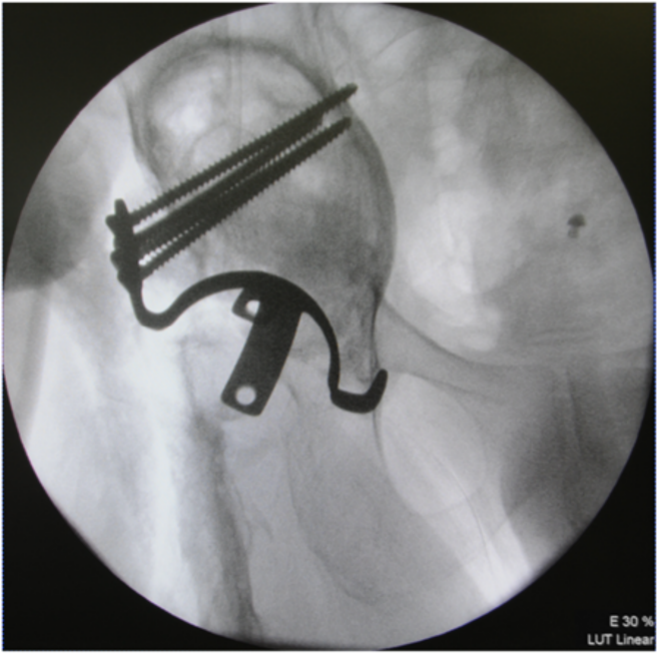
**Intra-operative photofluorography showing implantation of an acetabular roof reinforcement plate.**

**Figure 6:**
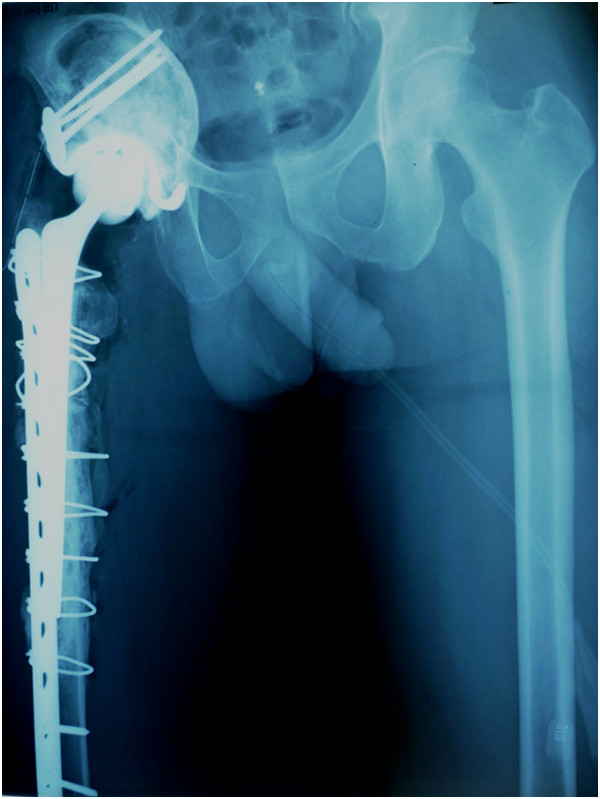
**X-ray showing reconstruction of the femur with a plate and a cable wire with cemented stem.**

A 42-year-old Japanese man was admitted to our hospital. He had sustained a right femoral neck fracture at the age of 19 years. At that time, he underwent osteosynthesis for the femoral neck fracture. The details of his operation were unclear, but he had developed an infection of the operative wound. There was no record of bacteriological examination performed at that time. One year later, when the patient was 20 years of age, the infection had resolved, and he underwent hemiarthroplasty of the hip joint with a bipolar cup. There was no record of that operation or the amount of damage to the acetabular cartilage. He had no particular problems following the procedure and was still able to surf as a hobby. He noticed a leg-length discrepancy, but he did not seek medical attention because there was no pain.Twenty years later, at age forty, the patient was injured while surfing. He felt a strong pain in the right femoral region and had difficulty walking. He was transported by ambulance to a nearby hospital. The diagnosis was Vancouver type B2 peri-prosthetic fracture of the femur. However, because the prosthesis had migrated, there was a massive bone defect of the acetabular roof on the pelvic side (Figure 1) that had resulted in the leg-length discrepancy.The plan was to treat only the peri-prosthetic fracture because the hospital had no bone bank. The implant was removed using a posterolateral approach and an extended trochanteric osteotomy. He underwent a new hemiarthroplasty of the hip joint with a modular neck and long stem. He also underwent osteosynthesis of the femur using a plate and hook for trochanteric fixation (Figure 2). The femoral shaft fracture achieved bone union, and his walking ability recovered to his previous level.He was later admitted to our hospital for reconstruction of the acetabular roof. He did not complain of coxalgia; however, his physical examination showed that the leg-length discrepancy was approximately 4.5cm. His Japanese Orthopedic Association (JOA) hip joint function score was 83. Three-dimensional computed tomography showed a massive American Academy of Orthopedic Surgeons type II cavitary bone defect of the acetabular roof (Figure 3).We planned to reconstruct the acetabular roof using an alloimplant for bone transplantation and a reinforcement plate for the acetabular roof, and to exchange the modular neck to correct the leg-length discrepancy. The operation was performed in our hospital using a posterior approach. We attempted to remove the modular neck to gain access to the acetabular side; however, the hook of the plate prevented extraction of the neck. We therefore removed the plate and planned to remove the modular neck, but it was adhered to the stem side. We therefore removed all implants using an extended trochanteric osteotomy. We developed the acetabulum and placed two alloimplants of femoral head bone into the weight-bearing region of the acetabular roof using an impaction bone graft method. We then implanted an acetabular roof reinforcement plate (Figures 4 and 5) and a cemented polyethylene cup at the site of the original acetabulum. Because reduction of the femur was difficult, we performed a subtrochanteric osteotomy of approximately 3cm. We reconstructed the femur using a plate and cable wire as well as a cemented stem (Figure 6). At the end of the operation, the patient’s leg-length discrepancy was 2cm; the shorter limb was the lower limb on the operated side.

**Figure 7:**
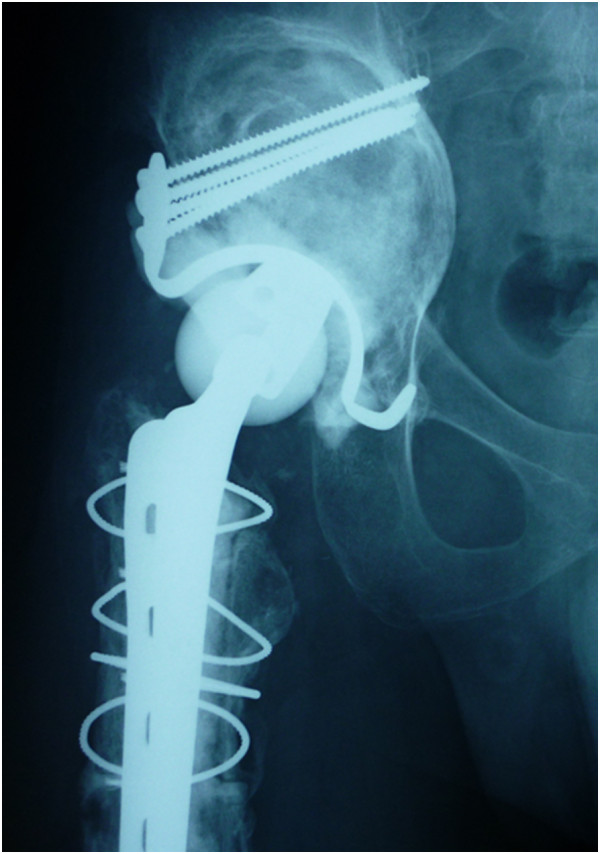
**Sample X-ray taken 18 months post-operatively showing complete union of the transplanted bone.**

The patient remained non-weight-bearing for 6 weeks post-operatively. He did not develop complications such as a dislocation or infection. At post-operative day 28, he was discharged from the hospital with a crutch.Eighteen months post-operatively, he had no coxalgia. His physical examination showed that his leg-length discrepancy was approximately 2cm, but he did not limp and he experienced no interference with his activities of daily living. His JOA functional test score was 100 points. X-rays showed complete union of the transplanted bone (Figure 7). The patient returned to his previous work and had no difficulties with everyday activities.

## Discussion

Treatment of femoral neck fracture in a young adult is usually accomplished by osteosynthesis. However, it is difficult to treat a patient with femoral head avascular necrosis, infection, or osteomyelitis.

After the infection subsided in our patient, we felt our only treatment option was hemiarthroplasty of the hip joint using a bipolar cup or total hip arthroplasty. For a femoral neck fracture without infection, hemiarthroplasty of the hip joint using a bipolar cup is reportedly not different from total hip arthroplasty [1]. We obtained satisfactory results with hemiarthroplasty of the hip joint using a bipolar cup [2]. However, this procedure can result in migration of the head and poor long-term results. Nakata *et al.* [3] reported a migration rate of 77% after hemiarthroplasty of the hip joint using a bipolar cup for secondary hip joint osteoarthrosis with acetabular dysplasia, as well as a higher migration rate in younger patients and those with a center-edge angle <0°. Tsumura *et al.* [4] found that patients who had hemiarthroplasty of the hip using a bipolar cup for avascular necrosis of the femoral head were categorized as Ficat stage II or III, whereas patients with avascular necrosis of the femoral head who were classified as stage IV had migration. Furthermore, when patients age 65 years or older or ambulatory patients age 75 years or older sustain a displaced femoral neck fracture, surgeons can expect total hip replacement to be better than hemiarthroplasty for the treatment of hip joint pain and erosion of the acetabular roof.

Total hip arthroplasty should be performed for a lesion on the acetabular roof, such as in rheumatism and osteoarthritis [5]. In our patient, in whom the articular cartilage of the acetabulum was mostly normal and who had good movement of the outer head, hemiarthroplasty of the hip joint using a bipolar cup was sufficient. However, when the articular cartilage of the acetabulum is damaged, total hip arthroplasty, rather than hemiarthroplasty with a bipolar cup, should be used to prevent migration. The presence or absence of cartilage damage should determine the approach to treatment in these cases.

## Conclusions

The treatment for a femoral neck fracture in a young adult is usually accomplished with osteosynthesis; however, difficulties may arise if there is accompanying infection, osteomyelitis, or femoral head avascular necrosis. When the acetabular articular cartilage is damaged, a bipolar cup should not be used; instead, total hip arthroplasty should be used to prevent migration. The presence or absence of cartilage damage should determine the approach to treatment in these cases.

## Consent

Written informed consent was obtained from the patient for publication of this case report and any accompanying images. A copy of the written consent is available for review by the Editor-in-Chief of this journal.
